# Characteristics and associated factors with sports injuries among children and adolescents

**DOI:** 10.1590/bjpt-rbf.2014.0059

**Published:** 2014

**Authors:** Franciele M. Vanderlei, Luiz C. M. Vanderlei, Fabio N. Bastos, Jayme Netto, Carlos M. Pastre

**Affiliations:** 1 Laboratório de Fisioterapia Desportiva, Departamento de Fisioterapia, Faculdade de Ciências e Tecnologia, Universidade Estadual Paulista, UNESP, Presidente Prudente, SP, Brazil; 2 Laboratório de Fisioterapia Desportiva, Programa de Pós-Graduação em Fisioterapia, Faculdade de Ciências e Tecnologia, UNESP, Presidente Prudente, SP, Brazil; 3 Programa de Pós-Graduação em Patologia Experimental, Universidade Estadual de Londrina, UEL, Londrina, PR, Brazil

**Keywords:** child, adolescent, traumatism in athletes, risk factors, rehabilitation

## Abstract

**BACKGROUND:**

The participation of children and adolescents in sports is becoming increasingly common, and this increased involvement raises concerns about the occurrence of sports injuries.

**OBJECTIVES:**

To characterize the sports injuries and verify the associated factors with injuries in children and adolescents.

**METHOD:**

Retrospective, epidemiological study. One thousand three hundred and eleven children and adolescents up to 18 years of age enrolled in a sports initiation school in the city of Presidente Prudente, State of São Paulo, Brazil. A reported condition inquiry in interview form was used to obtain personal data and information on training and sports injuries in the last 12 months. Injury was considered any physical complaint resulting from training and/or competition that limited the participation of the individual for at least one day, regardless of the need for medical care.

**RESULTS:**

The injury rate per 1000 hours of exposure was 1.20 among the children and 1.30 among the adolescents. Age, anthropometric data, and training characteristics only differed with regard to the presence or absence of injuries among the adolescents. The most commonly reported characteristics involving injuries in both the children and adolescents were the lower limbs, training, non-contact mechanism, mild injury, asymptomatic return to activities, and absence of recurrence.

**CONCLUSIONS:**

The injury rate per 1000 hours of exposure was similar among children and adolescents. Nevertheless, some peculiarities among adolescents were observed with greater values for weight, height, duration of training, and weekly hours of practice.

## Introduction

The participation of children and adolescents in the practice of physical activities and sports has increased in recent decades^1^. It is estimated that 30 to 45 million individuals between six and 18 years of age participate in sports^2^.

The practice of sports provides benefits to the cardiopulmonary, musculoskeletal, and endocrine systems. Sports lead to improvements in motor skills and daily habits as well as the acquisition of dexterity, exerting an influence on the social and psychological aspects of practitioners^1^^-^ 4.

However, constant exposure to repetitive motor actions and excessive load poses the risk of injury^5^^,^ 6. Indeed, Adirim and Barouh^7^ reported that when children practice a sport, they are exposed to injury and, in this context, several risk factors can be considered, such as musculoskeletal immaturity, obesity, and characteristics of training. Thus, it is important to identify the factors associated with injury to establish preventive strategies.

The first step to knowledge regarding such occurrences is to carry out investigations of an epidemiological nature. Thus, the aim of the present study was to characterize the sports injuries and verify the associated factors with injuries in children and adolescents.

## Method

### Subjects

A total of 1311 student athletes (939 males and 372 females) enrolled with the City of Presidente Prudente Municipal Sports Department (State of São Paulo, Brazil) in the sports modalities of athletics, basketball, football, soccer, gymnastics, karate, kung fu, swimming, table tennis, and volleyball were randomly selected for participation in the present study. The volunteers were divided into two groups: children (n=509) aged up to 12 years of age and adolescents (n=802) aged 12 to 18 years, based on the classification set by Brazil's Child and Adolescent Statute enacted in 1990^8^. All volunteers were amateurs and beginners in the practice of sports.

This study received approval from the Human Research Ethics Committee of *Universidade Estadual Paulista* (UNESP), Presidente Prudente, SP, Brazil, under process number 08/2010 and all volunteers signed an informed consent form.

### Study design and field procedures

The data were collected through individual interviews addressing the occurrence of injuries and respective characteristics in the previous 12 months of training and/or competition. To avoid interfering in the normal dynamics and routine of the sport, the volunteers were approached either prior to or following training sessions.

A reported condition inquiry was used, which has been used for the acquisition of information on general health status in specific populations due mainly to its applicability and objectivity^9^^-^ 12. A pilot study was first conducted with 200 individuals to test the applicability of this instrument, the results of which demonstrated adequate comprehension of the questions on the part of the respondents.

Data were collected individually in interview form by a single examiner familiarized with the instrument. Pastre et al.^9^ suggests this procedure due to the different degrees of understanding regarding the annotation of answers on the part of interviewees. Information was provided by the volunteers as well as their coaches and/or parents/guardians, as suggested by Pereira^13^ for the acquisition of data related to health conditions.

### Injury Reporting

Sports injury in the present study was defined as any physical complaint resulting from training and/or competition that limited the participation of the individual for at least one day, regardless of the need for medical care. This definition has been employed in previous studies^14^^,^ 15.

The inquiry addressed personal data, such as gender, age, weight, height, and duration of training in years, which were considered the independent variables. Body mass was determined using a Filizola scale with a precision of 0.1 Kg and height was determined using a Sanny portable stadiometer with millimeter measurements. For these measurements, the volunteers were barefoot and wore light clothing.

The inquiry addressed sports injuries, such as the anatomic site affected, injury mechanism, when the injury occurred, severity of the injury, return to normal activities, and recurrences. To facilitate the identification of the anatomic site of the injury, an illustration of the human body was shown, on which the subject marked the region of the body referring to the sensation of pain or musculoskeletal discomfort. The determination of the injury mechanism consisted of the volunteer's perception regarding the contact or exact action performed when signs and symptoms of an acute episode emerged and/or the type of activity in which such manifestations were accentuated. This variable was divided into direct contact and non-contact^16^^-^ 18. The moment of occurrence of the injury was analyzed based on the specific phase of training or competition. The severity of the injury was classified based on the *National Athletic Injury Reporting System*, which classifies sports injuries based on the time the athlete spends away from the sport for recovery^19^^,^ 20. The determination of the return to normal activities addressed whether or not this event occurred and whether the return to the sport without any alterations in normal training occurred with or without signs and/or symptoms. The recurrence of injury was investigated to determine whether injury had occurred on other occasions and in the same anatomic site on other occasions^19^.

### Organization and description of categories of variables

To facilitate the analysis and presentation of the results, the variables were subdivided into categories based on the most expressive clusters of results without affecting the essence of their origin or the conclusions of the study. Regarding anatomic site of pain or discomfort, the questionnaire listed 20 bodily regions, which were grouped into the following segments: upper limbs, lower limbs, and trunk.

The following two injury mechanisms were considered: i) injury due to direct contact caused by a single traumatic incident, such as a fall or collision with an opponent^16^^-^ 18; ii) non-contact injuries stemming from aspects inherent to the sport itself, such as short and long-distance runs, rapid changes in movement, jumps, and landing^16^^-^ 18.

Severity was categorized as mild injury (1 to 7 days away from sport), moderate injury (8 to 21 days away from sport) or severe injury (more than 21 days away from sport or permanent injury)^19^^,^ 21.

### Statistical analysis

Descriptive statistics were used for the analysis of the profile of the population and description of the variables. The results were expressed as mean and standard deviation values, percentages, and absolute numbers. The odds ratio (OR) test with a 95% confidence interval (CI) was used to determine whether the presence/absence of injury was associated with age group and gender. The Kolmogorov-Smirnov was used to test the normality of the data. Student's t-test for non-paired data was used in cases of normal distribution (height in the group of children) and the Mann-Whitney test was used for cases in which normal distribution was not found (all other independent variables). Goodman's test for contrasts between and within multinomial populations was used to test associations between the characteristic of the group of variables to be analyzed and anatomic site, injury mechanism, when the injury occurred, severity, return to normal activities, and recurrence. The frequency of injury was calculated by the number of athletes interviewed who reported injury in the period x 100.000/total number of athletes interviewed. The risk of injuries for injured athlete was calculated by the number of athletes interviewed who reported injury in the period/number of injuries. The injury rate per 1000 hours of exposure was calculated by numbers of injuries divided by the number of exposure hours multiplied by 1000. The statistical analyses were conducted using the *Minitab* program, version 13.3, with the significance level set at 5% (p<0.05).

## Results

Among the group of children, the mean age was 10.46±1.61 years, weight was 41.28±11.34 kg, height was 1.46±0.10 m, duration of training was 1.60±1.04 years, and weekly hours of practice were 2.90±2.04. Among the group of adolescents, mean age was 14.55±1.36 years, weight was 58.95±12.15 kg, height was 1.46±0.10 m, duration of training was 2.68±2.10 years, and weekly hours of practice were 5.20±3.72.

Among the 1311 interviewees, 234 athletes reported a total of 261 injuries, corresponding to more than one injury per injured athlete. Statistically significant differences were found in the frequency distribution of injuries between the two age groups, with the adolescents demonstrating a greater risk of injury than the children (OR: 1.97; 95% CI: 1.44-2.70). As no significant gender differences were found among either the children (OR: 0.82; 95% CI: 0.45-1.50) or the adolescents (OR: 0.98; 95% CI: 0.68-1.41), the analyses were performed without gender distinctions. The frequency of injury was 12% among the children and 21% among the adolescents. The frequency per injured athlete was 14% among the children and 25% among the adolescents. The injury rate per 1000 hours of exposure was 1.20 among the children and 1.30 among the adolescents (Table 1).

**Table 1:** Mean values, followed by the standard deviation, and confidence interval of injury rate per 1000 hours of exposure and absolute (n) and relative (%) frequency of injured athletes, injuries reported and frequency of injury.

Variables	Groups	
	Children (n=509)	Adolescents (n=802)
Injury rate per 1000 hours of exposure	1.20±3.6 [0.89–1.52]	1.30±3.05 [1.09–1.51]
Injured athletes	62 (12.18)	172 (21.44)
Injuries reported	64 (12.57)	197 (24.56)
Injury risk	0.12	0.24
Injury risk per injured athlete	1.03	1.14
Frequency	12%	21%

Injury risk per athlete = total number of injuries divided by total number of athletes interviewed; injury risk per injured athlete = total number of injuries divided by total number of injured athletes; Injury rate per 1000 hours of exposure = numbers of injuries divided by the number of exposure multiplied by 1000.

Among the adolescents, weight, height, duration of training, and weekly hours of practice were associated with injuries, with higher median values for these variables among individuals affected by injuries than non-affected individuals (Table 2).

**Table 2:** Mean, standard deviation, median, and confidence interval values for anthropometric measures and training variables according to age group and occurrence of injury.

Variables	Groups	Injured	Non-injured	*p*-value
Weight (kg)	Children	43.3±11.03 (42.80) [40.59–46.10]	40.99±11.37 (39.20) [39.93–42.04]	0.09
	Adolescents	62.40±13.08 (60.30)* [60.57–64.23]	57.87±11.64 (56.40) [56.96–58.78]	0.0001
Height (m)	Children	1.48±0.10 (1.48) [1.46–1.51]	1.46±0.10 (1.46) [1.45–1.47]	0.10
	Adolescents	1.69±0.09 (1.70)* [1.68–1.71]	1.65±0.08 (1.66) [1.64–1.66]	0.0001
Duration of training (years)	Children	1.82±1.18 (1.00) [1.52–2.11]	1.57±1.02 (1.00) [1.47–1.66]	0.06
	Adolescents	3.44±2.52 (3.00)* [3.09–3.80]	2.44±1.90 (2.00) [2.30–2.59]	0.0001
Weekly hours of practice	Children	3.46±2.98 (2.00) [2.72–4.21]	2.82±1.86 (2.00) [2.65–3.00]	0.44
	Adolescents	6.63±4.36 (6.00)* [6.02–7.24]	4.75±3.38 (4.00) [4.49–5.01]	0.0001

Kolmogorov-Smirnov normality test

*Statistically significant difference in relation to non-injured athletes; The Mann-Whitney test was used to compare medians between injured and non-injured athletes for height, weight, duration of training, and weekly hours of practice in adolescents.

Table 3 shows that the lower limbs had a significantly greater number of injuries in both groups in comparison to the upper limbs and trunk. A greater number of injuries occurred during training in both groups. Among the adolescents, the non-contact mechanism (72.59%) differed significantly from the direct contact mechanism (24.36%).

**Table 3:** Absolute (n) and relative (%) frequency of anatomic site, when injury occurred, and injury mechanisms.

Variables	Groups	
	Children (n=509)	Adolescents (n=802)
Anatomic Site		
Upper limbs	12 (18.75)	42 (21.32)
Lower limbs	48 (75.00)*	131 (66.50)*
Trunk	4 (6.25)	24 (12.18)
Total	64 (100)	197 (100)
When injury occurred		
Training	59 (92.18)†	160 (81.22)†
Competition	5 (7.82)	37 (18.78)
Total	64 (100)	197 (100)
Mechanism		
Direct contact	27 (42.19)	48 (24.36)
Non-contact	37 (57.81)	149 (75.64)‡
Total	64 (100)	197 (100)

Goodman's test for contrasts between and within multinomial populations

*Difference in relation to upper limbs and trunk

†Difference in relation to competition

‡Difference in relation to direct contact.

Table 4 shows that, in both groups, a greater frequency of mild injury was found in comparison to moderate and severe injury. The majority of the children returned to their normal activities asymptomatic, whereas similar proportions of adolescents returned to their normal activities with and without the presence of signs and/or symptoms. A statistically significant difference was found between the absence and presence of recurring injury among the adolescents.

**Table 4:** Absolute (n) and relative (%) frequency of injuries according to severity, return to activities, and recurrence.

Variables	Groups	
	Children (n=509)	Adolescents (n=802)
Severity		
Mild	56 (87.50)*	159 (80.71)*
Moderate	4 (6.25)	20 (10.15)
Severe	4 (6.25)	18 (9.14)
Total	64 (100)	197 (100)
Return to normal activities		
Asymptomatic	50 (78.12)†	111 (56.35)
Symptomatic	14 (21.88)	86 (43.65)
Total	64 (100)	197 (100)
Recurrence		
No	37 (57.82)	133 (67.51)‡
Yes	27 (42.18)	64 (32.49)
Total	64 (100)	197 (100)

Goodman's test for contrasts between and within multinomial populations

*Difference in relation to moderate and severe injury

†Difference in relation to symptomatic return

‡Difference in relation to recurrence.

## Discussion

The investigation into injuries associated with the different sports practiced in Brazil among individuals under 18 years of age demonstrates that the injury rate per 1000 hours exposure does not appear to show significant differences between children and adolescents, which does not allow for a comparison of characteristics of injuries between the groups. Despite the correction in relation the exposure of each athlete, each group studied seems to have particular characteristics regarding the occurrence of injuries. In the group of adolescents the occurrence of injuries was associated with age, anthropometric data and training variables, and among the children was found random distribution for these variables.

The frequency of injury was 12% among the participants aged six to 11 years and 21% among those aged 12 to 18 years. Conn et al.^22^ estimate that 22% of injuries among individuals aged five to 24 years are sports-related. A study carried out in Norway reports this figure to be around 17%^23^. However, when the correction for exposure of the athlete is applied, mean values of the injury rate per 1000 hours exposure are confirmed to be similar for both groups, in contrast with the findings of Knowles et al.^5^, who reported a rate of 1.41 for children under 14 years and 2.52 for individuals 18 years of age.

Each group studied seems to have particular characteristics regarding the occurrence of injury. The results of the present study demonstrate that the frequency of injury increased in the adolescents, which is in agreement with findings described in previous studies^24^ ^,^ ^25^. The reasons for this are related to the greater involvement in sports with the advance in age, in addition to the characteristics of training such as high intensities of physical stimuli and inadequate recovery time^26^.

Regarding anthropometric and training characteristics, injuries were significantly associated with intrinsic and extrinsic risk factors only in the group of adolescents. The median values for weight, height, duration of training, and weekly hours of practice were higher among the athletes with a recent history of injury. Studies report a greater frequency of injury among heavier and taller adolescents due to the generation of a greater magnitude of forces absorbed by soft tissues and joints; the greater dynamism and collision force also contribute toward the occurrence of injury in this specific population^27^^-^ 29. In the present study, adolescents with a greater duration of training and greater number of weekly hours of practice reported more injuries. The increase in exposure may be related to an increased risk of injury due to repetitive and cumulative trauma, as reported by Turbeville et al.^30^.

Among the children, no specific profiles were observed for the variables studied with regard to the presence or absence of injury, which does not allow the establishment of associations with the occurrence of injury in this age group. Among the adolescents, however, there was a tendency toward an increase in confidence interval values among the injured individuals in comparison with the non-injured individuals. Thus, the results of the present study indicate the need for specific care when exceeding three years of practice within a given sport and six hours of practice per week.

Regarding the anatomic site, there was a predominance of injuries in the lower limbs, especially the knees and ankles. Sharma et al.^31^ found that the frequency of sports injuries among children up to 12 years of age was 43.8% in the upper limbs, 34.5% in the lower limbs, and 16% in the head, which differs from the findings of the present study. However, in a study involving adolescents up to 16 years of age practicing 15 different sports, Hootman et al.^17^ found that the lower limbs were the most affected during both training and competitions, which is in agreement with the results of the present study. This finding may be explained by the fact that sports generally involve common activities, such as running, jumping, and rapid changes in direction, which directly affect the lower limbs and increase the risk of injury in this anatomic site^32^.

Most of the injuries occurred during the training period. This finding may be related to the greater exposure of the present sample during training sessions, as participation in competitions is far more limited. However, there is no consensus on this issue. A number of authors report that injuries are more common during competitions^17^^,^ 30. Moreover, Rechel et al.^32^ report similar proportions of injuries occurring during competition (51.5%) and training (48.5%).

Non-contact injuries were more commonly reported by adolescents than children. Thus, the biomechanical aspects of the specific actions and/or metabolic expenditures involved in the sport seem to become more pronounced with age^17^. This underscores the importance of addressing issues linked to the biomechanics and physiology of effort as causal agents of injury^17^.

However, descriptively, both children and adolescents showed the non-contact mechanism as being the most frequent. Ribeiro and Costa^26^ describe that high incidence rates of non-contact injuries can indicate that athletes had insufficient time of preparation for the demands of training and/or there was not sufficient time for the recovery of the stimuli during training. Thus, special attention should be given to the type of training and the biomechanical and physiological characteristics trained to prevent this type of injury.

Regarding severity, there was a predominance of mild injuries. Rechel et al.^32^ found that the majority of injuries resulted in at least one week away from normal activities, whereas 30.3% of injuries led to one to three weeks away, 6.8% resulted in more than three weeks away from activities, without ending the athletes' career and 10.4% of injured athletes did not return to either the season or their career. The fact that the majority of injuries in the present study were mild may be explained by the characteristics of the sample, which was mostly made up of individuals in the sports initiation phase, who experience lesser training intensity and physical contact in comparison to the training category, as suggested by Rechel et al.^32^.

There was a greater frequency of asymptomatic return to normal activities, which is of fundamental importance to children and adolescents as they are in a period of growth and development. Therefore, along with adequate musculoskeletal rehabilitation, instructions regarding the prevention of further injury should be emphasized^33^.

Descriptively, the percentage of recurrence in children was 42% and in adolescents was 32%, being considered high for several types of injury. Powell and Barber-Foss^34^, who reported a recurrence risk of only 10% (range: 8.4% to 13.9%) among the different sports analyzed, posed the hypothesis that this may be an indicator of the positive influence of the participation of injured players in prevention programs aimed at minimizing the likelihood of further injury. However, it should be pointed out that the sample in the present study was not submitted to any type of specific preventive work, which may explain the high percentage found in this population. Thus, the importance of preventive programs on the recurrence of injury is evident.

The data collection instrument has been used for the acquisition of information on high-performance athletes^9^ and there are no records of its use on children and adolescents in Brazil. However, the analysis tool and approach involved the utmost care, as described in the Methods section, to ensure maximum reliability. Thus, the reported condition inquiry appears to be an excellent way to record epidemiological data with ease and coherence.

Nevertheless, the retrospective design constituted a limitation of the present study, as data may have been lost in the time interval analyzed and the actual magnitude of the injuries may have been underestimated by recall bias. Another limitation found was the lack of registration of exposure in hours separated by different periods, training and competition, precluding further analysis about the time the injury occurred.

Joint actions uniting health and sports sciences, specially physical therapy^35^^,^ 36, should be encouraged for the establishment of strategies aimed at offering greater safety to beginners in the practice of any sports modality. Actions of this nature may have a positive impact on health, especially among children and adolescents, as well as consequences in the social realm.

## Conclusion

The injury rate per 1000 hours of exposure was similar among children and adolescents, whereas the frequency of injury without exposure correction overestimated the occurrence of injury in adolescents. Nevertheless, some peculiarities among adolescents were observed with greater values for weight, height, duration of training, and weekly hours of practice. The following characteristics of injury predominated in both groups: lower limbs, training period, the non-contact mechanism, mild injuries, and asymptomatic return to normal activities. Furthermore, the presence of recurrence was considered high for both groups.
